# Gem-diol and Ketone Crystal-to-crystal Transition Phenomena

**DOI:** 10.1038/s41598-017-13596-6

**Published:** 2017-10-18

**Authors:** Zhang Bo, Chen Sitong, Guo Weiming, Zhang Weijing, Wang Lin, Yang Li, Zhang Jianguo

**Affiliations:** State Key Laboratory of Explosion Science and Technology, Beijing Institute of Technology, Beijing, 100081 China

## Abstract

The generally thought unstable diol compound tetrazyl gem-diol (1, H_2_DTMdiol·2H_2_O), was firstly obtained in crystalline form by culturing the filtrate for ten days after acidification and filtration of aqueous solution of potassium salt of ketone (2, [K(HDTMone)·2H_2_O]_n_). The stability of this novel gem-diol compound is found owning to the hydrogen bonds with lattice water molecules and electrophilic tetrazolyl groups. Meanwhile, the undissolved ketone (3, H_2_DTMone) was separated during the filtration in the process of gem-diol compound production. Surprisingly, the crystal-to-crystal perfect transition phenomena from gem-diol (1) to ketone (3) were firstly observed after heating up to 120^ °^C as evidenced by X-ray single crystal diffraction and powder X-ray diffraction. These results found here might open new revenues for methylene oxidation and alkanediol chemistry.

## Introduction

**Figure 1:**
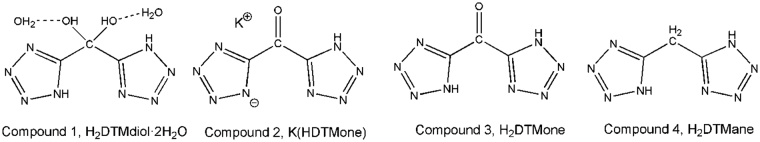
The structures of compound 1 to 4.

A very rare phenomenon was observed by chance at the attempt to develop new HEDMs (high-energy-density materials). Introducing effective oxygen atoms is a very important and simple way to design new HEDMs with better detonation properties since nitration reaction is the most common method in this field. It is obvious that oxidation of -CH_2_- can introduce effective oxygen atoms. So the oxidation of di(1H-tetrazol-5-yl)methane (4, H_2_DTMane) was conducted using potassium permanganate as oxidizing agent. And the crystalline tetrazyl gem-diol (-C(OH)_2_-) compound, di(1H-tetrazol-5-yl)methanediol dihydrate (1, H_2_DTMdiol·2H_2_O) (see Fig. 1), was obtained for the first time.

The electron withdrawing groups in the previously reported crystalline gem-diol compounds include trichloromethyl, pyridyl, ester group etc except tetrazyl^1–7^. This discovery is very important to gem-diol chemistry since none of crystalline tetrazyl gem-diol compound has been reported until now.

As known, compounds based on azoles are ubiquitous and have attracted much attention owing to their variety of applications in medicine, biology and material science^8–10^. Among them, tetrazole and its derivatives have drawn researchers’ attention in developing HEDMs due to their high nitrogen content resulting the enrichment of high energy bonds such as N-N (160 kJ·mol^−1^) and N = N (418 kJ·mol^−1^)^11,12^. The pioneer research teams such as KlapÖtke’s, Shreeve’s, and many other groups have contributed considerable studies^13–19^. The key question in new HEDMs development is the balancing of high-energy and insensitivity. One effective method is increasing oxygen balance properly by introducing oxygen-containing functional groups such as -NO_2_, -NH-NO_2_ and -OH. It is important to verify whether the introduction of carbonyl group (-C( = O)-) works well or not.

**Figure 2:**
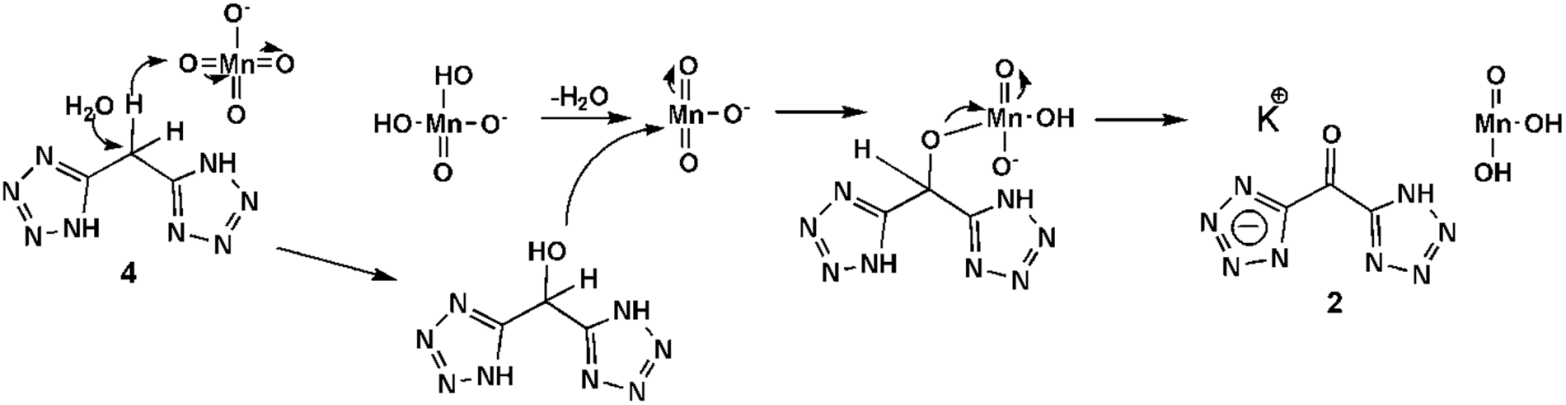
The supposed mechanism for the autocatalysisoxidation by potassium permanganate.

Therefore, we start from di(1H-tetrazol-5-yl)methane (4, H_2_DTMane) to synthesize target compound. The synthesis of H_2_DTMone was optimized. Then, excess inorganic oxidant potassium permanganate was used to achieve the transformation of methylene (-CH_2_-) to carbonyl (-C( = O)-) bridging group. The possible mechanism for the autocatalysis oxidation using potassium permanganate was given in Fig. 2 according to relevant literature^20^. The oxidation process of the methylene group may go through two states including addition of water to -C(OH)H- group and dehydration with MnO_3_^−^ to -C( = O)-. The insoluble substance was proven to be manganese dioxide hydrate, while the oxidation product potassium di(1H-tetrazol-5-yl)methanone dihydrate (2, [K(HDTMone)·2H_2_O]_n_) was dissolved in the aqueous solution. Therefore, it is convenient to collect compound 2 by evaporation after filtration.

White powder di(1H-tetrazol-5-yl)methanone (3, H_2_DTMone) can be obtained after acidification and filtration of solution of compound 2, since compound 3 hardly dissolves in water at room temperature. The crystalline tetrazyl gem-diol (-C(OH)_2_-) compound, di(1H-tetrazol-5-yl) methanediol dihydrate (1, H_2_DTMdiol·2H_2_O), was grown from the above filtrate in ten days. It is a regular transparent crystal.

**Figure 3:**
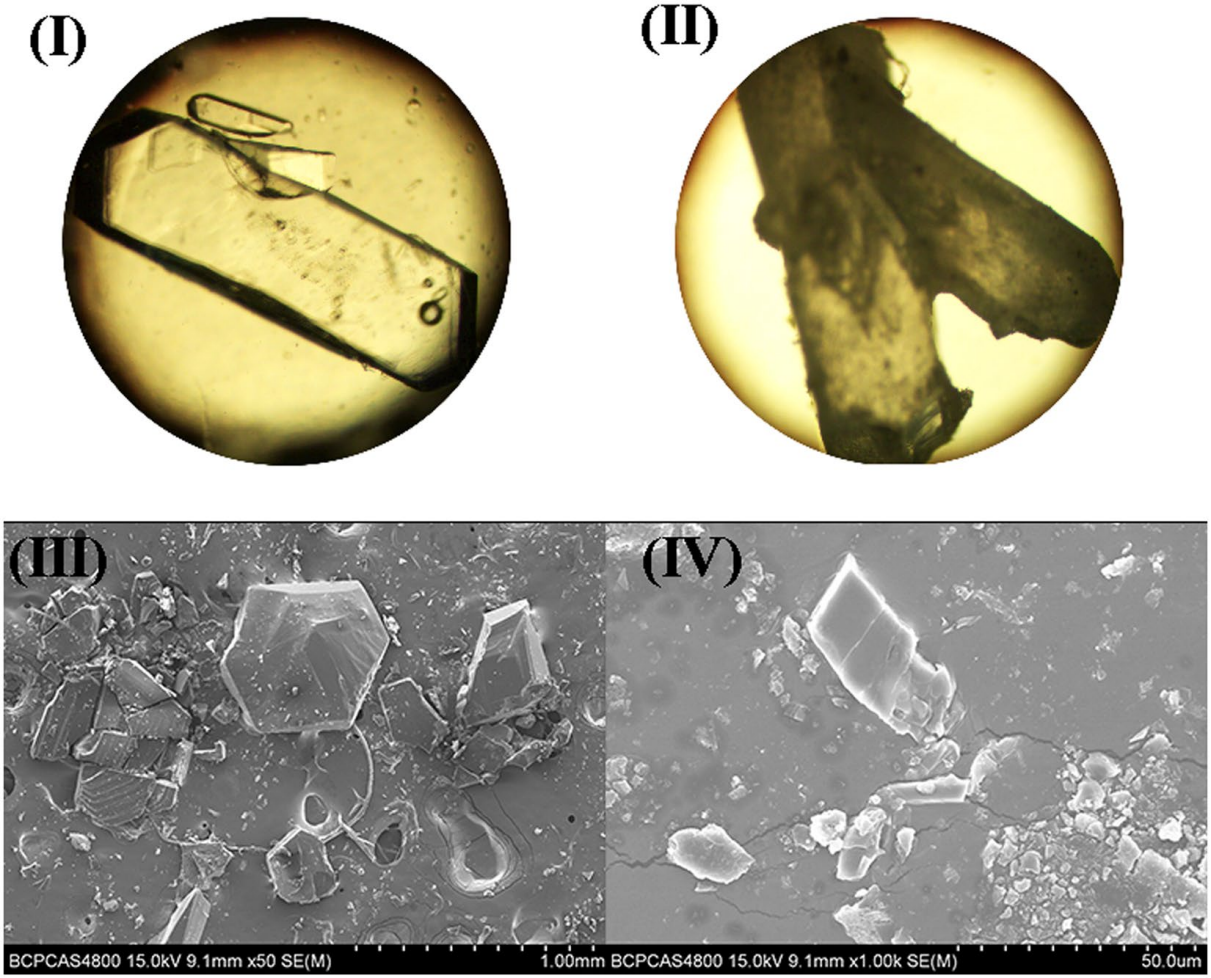
(**I**) and (**III**) crystal appearance before transition (compound 1); (**II**) and (**IV**) crystal appearance after transition (compound 3).

Surprisingly, crystal-to-crystal transition occurred from crystal of compound 1 to crystal of compound 3 was firstly observed after heating up to 120 °C as evidenced by X-ray single crystal diffraction and powder X-ray diffraction (PXRD, Figures S11 and S12). Although the transformed crystal is cracked, the much smaller but suitable crystal size of compound 3 could be picked out for X-ray single crystal diffraction measurement. Figure 3 shows the crystal appearances before and after transition, in which (II) was derived from (I). And crystal suitable for X-ray single crystal diffraction measurement could be picked out from (II). (III) and (IV) are electron-microscope photos before and after the transition, for which the smaller size crystals of compound 1 were grown. It can be seen in both electron-microscope photo and optical microscope photo that the crystal of compound 1 is irregularly hexagon block, while the crystal appearance after transition (compound 3) is just irregular block in much smaller size. To our knowledge, this phenomenon is so unusual that rarely reported in literatures.

As unstable intermediate compounds, gem-diol compounds are ubiquitous in hydrolysis reactions of aldehydes and ketones. Unfortunately, the structures in which two hydroxyl groups are linked by a carbon atom are generally photolabile and thermolabile. So it is quite challenging for researchers to study their specific characteristics. The stable gem-diol compounds are rarely reported, while that of tetrazyl gem-diol compounds are to our knowledge never reported before. Among these, ninhydrin and chloral hydrate were well-known and fully studied gem-diol compounds. Ninhydrin is generally used as chromogenic agent of amino acids^21,22^. And chloral hydrate is broad investigated in medical field^23,24^. However, the phenomenon of crystal-to-crystal transition in this kind of compounds is never reported until this work in the field of germinal diol chemistry. The crystalline compound 1 is dehydrated to compound 3, which still remains perfect crystalline state. Fortunately, we cultured crystals and carried out X-ray single crystal diffraction analysis which unambiguously confirmed the structures and these phenomena substantially.

**Figure 4:**
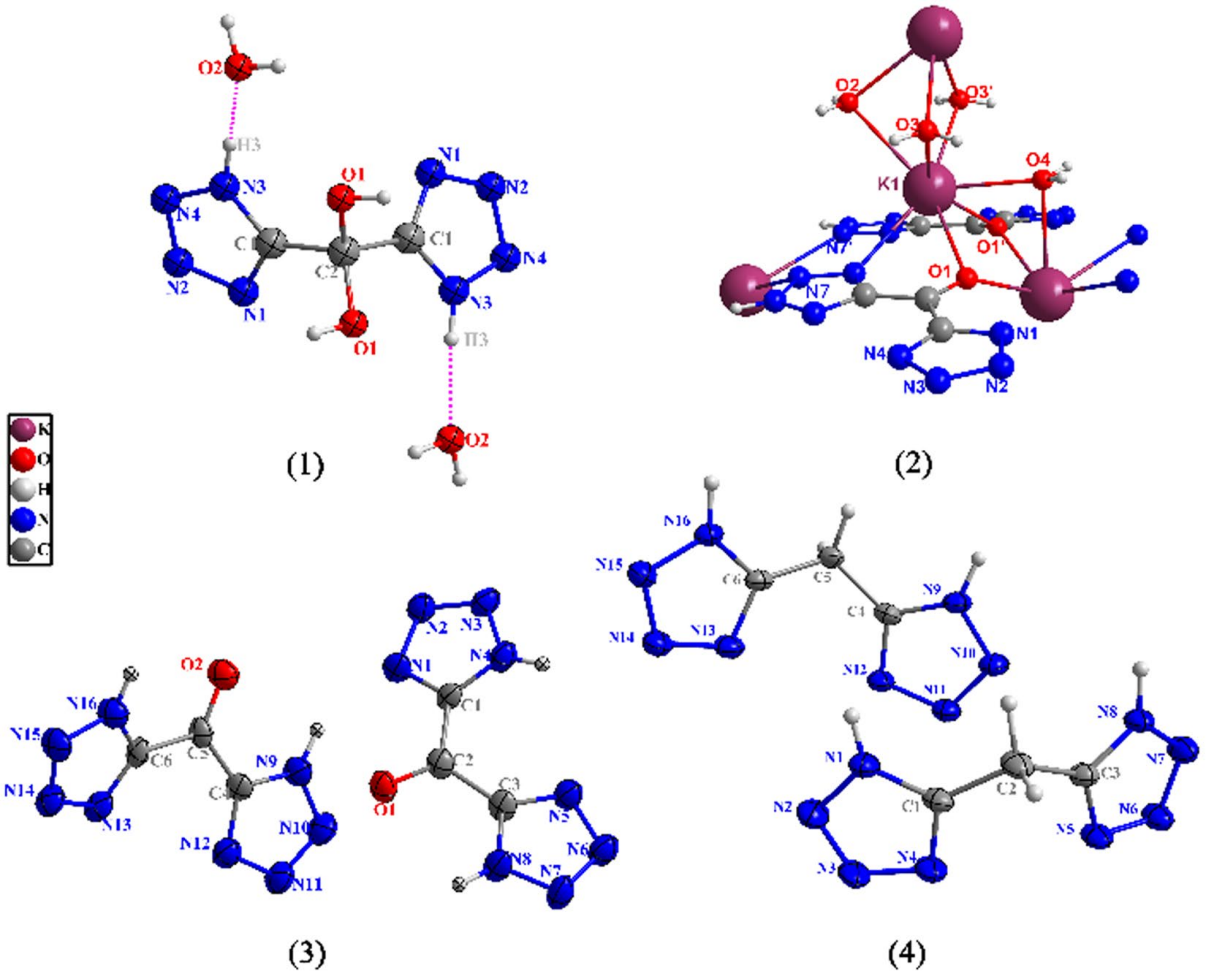
Thermal ellipsoid diagrams of the crystal structures of compound 1, 2, 3 and 4.

The structures of compound **1**, **2**, **3** and **4** were obtained by X-ray single crystal diffraction as shown in Fig. 4 and Table S1. Further information of the crystal-structure determinations have been deposited in the Cambridge Crystallographic Data Centre as supplementary publication No. 1516736, 1516734, 1439053, 1516733.

Compound 1 crystallizes as a dihydrate in the monoclinic space group C2/c with four molecules in one unit cell. The structure 1 is composed of two tetrazole rings connected with C1-C2-C1′, of which both the two bond lengths are the same as 1.519 Å and the bond angle is 110.4(2)°. The unique bond length of C2-O1 is 1.39 Å. The bond angle of ∠O1-C2-O1′ is 114.2°. The O2-tetrazole1-O1-C2 and O2′-tetrazole2-O1′-C2 fragment are almost planar, respectively. The dihedral angle formed between the planes of the two fragments is 75.14(3)°. The packing structure of compound 1 is shown in Fig. S5and S2. The molecules are connected by H-bonds to 3-D networks, which contributes to its higher dehydration temperature than that of 5-(5-Nitrotetrazol-2-ylmethyl) tetrazole monohydrate reported by Klapötke *et al*. (99 °C)^25^. And the latter only have H-bonds along b-axis.

**Figure 5:**
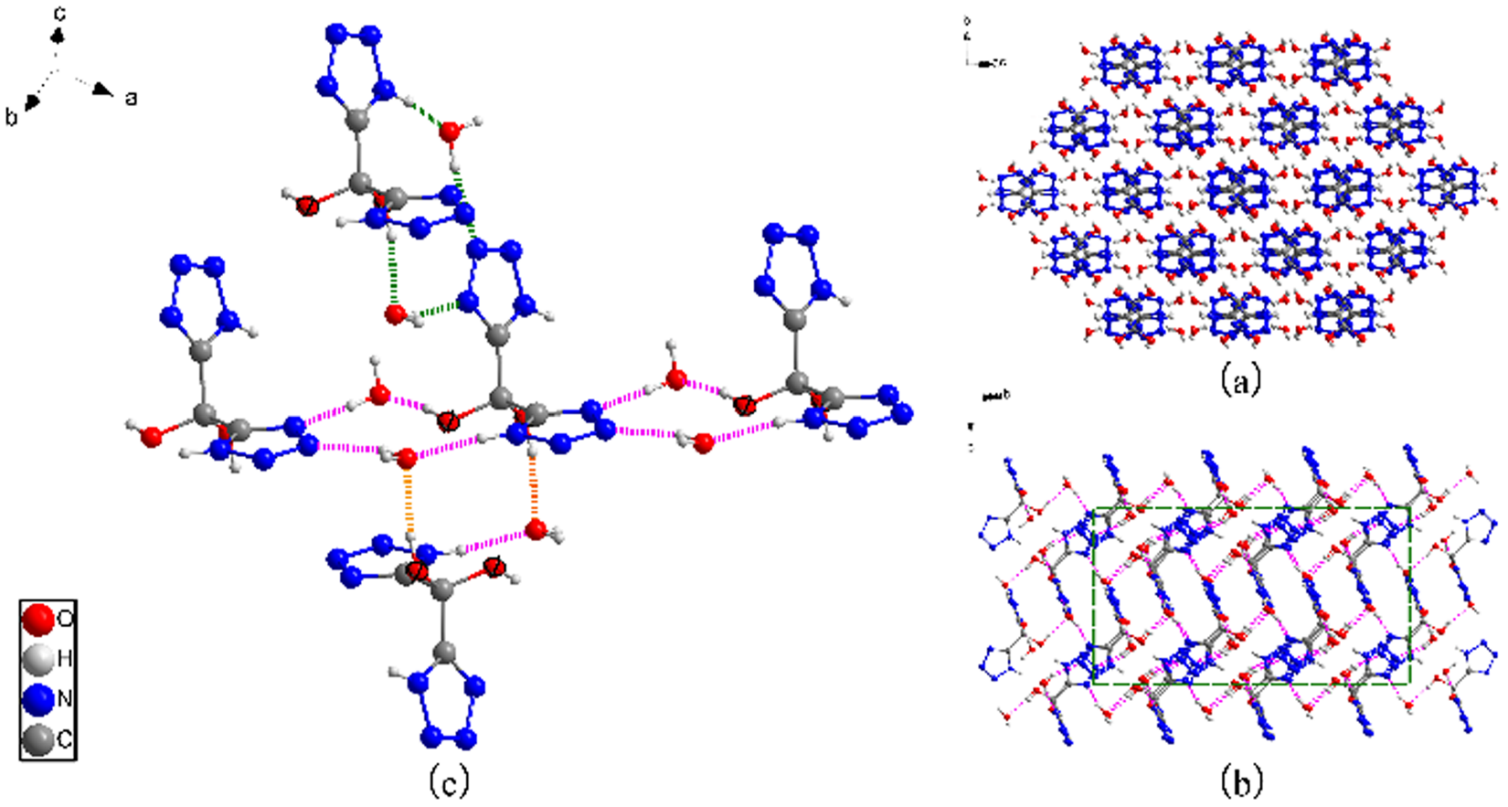
(**a**) The packing structure of compound 1; (**b**) The intermolecular hydrogen-bond networks, dashed lines indicate strong hydrogen bonding; (**c**) Detail with enlarged scale Hydrogen-bonding interaction of compound 1.

Compound **2** crystallizes in the monoclinic space group P21/m with four molecules in the unit cell. Compound **2** demonstrates coordination structure through the connection of K^+^ ions with carbonyl groups and water molecules. Each K^+^ ion is eight coordinated by four water molecules and the N8, N8′, O1, O1′ from two different di(1H-tetrazol-5-yl)methanone. A two-dimensional network of **2** (Figure S4) is formed by dimeric potassium tetrazolate dihydrate units. Due to its 2-D frameworks stabilizing the structure, compound 2 possesses a decomposition peak temperature of 205.1 °C higher than that of compound 3.

**Figure 6:**
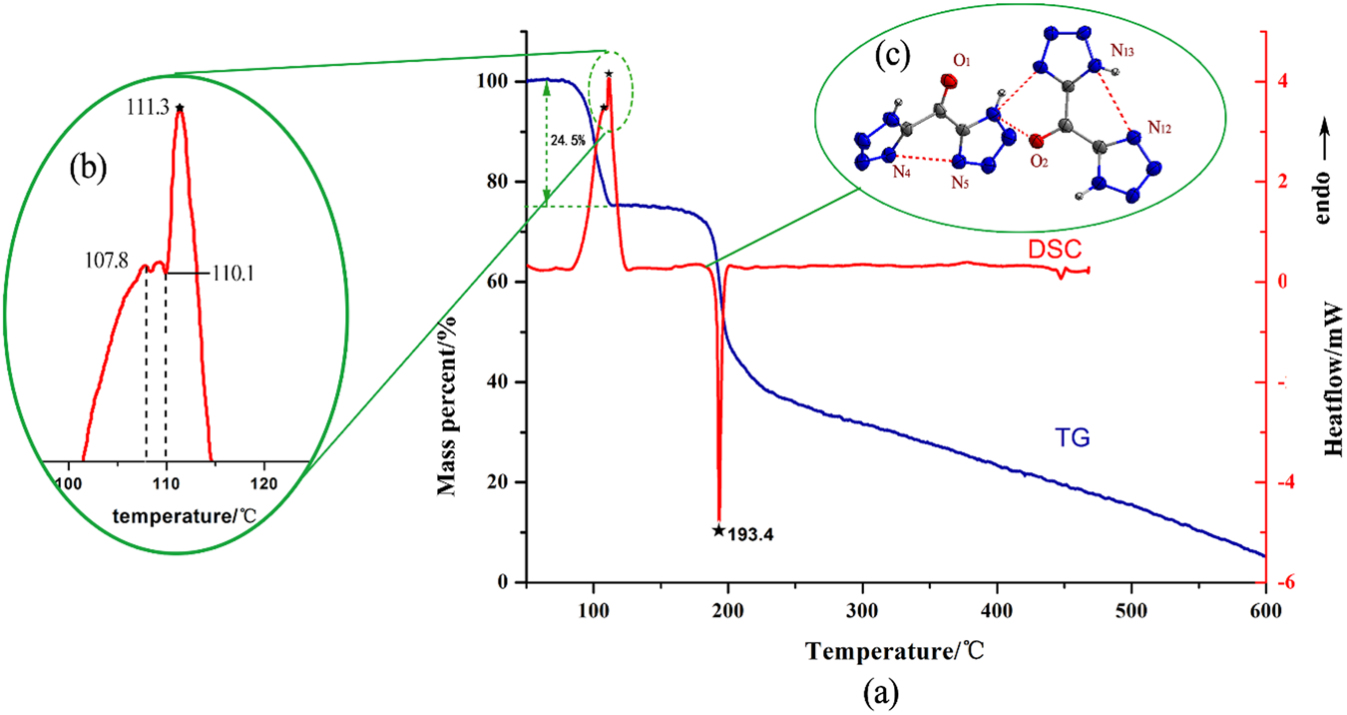
(**a**) DSC and TG curves of crystal 1; (**b**) Detail with enlarged scale of DSC measurement; (**c**) Molecular structure of compound 3.

Crystal **3** was obtained by the dehydration of crystal **1**, which was confirmed by single X-ray diffraction. Compound **3** crystallizes in triclinic space group P-1. As expected, after the methylene group of **4** was oxidized to carbonyl group, the density has also been significantly improved to 1.734 g·cm^−3^. The bond length of C2-O1 is 1.21 Å. The carbonyl bridged two tetrazole rings are not in the same plane with a torsion angle of ∠C4-C5-C6 of 118.5(5)°. There are few H-bonds in compound 3, which result in lower decomposition temperature (193.4 °C, Fig. 6). As comparison, the similar compounds di(1H-tetrazol-5-yl) methanone oxime and 5,5′-(hydrazonomethylene)bis(1H-tetrazole) reported by Shreeve *et al*.^26^ decompose at 288.7 °C and 247.6 °C respectively, whose structures are stabilized by much more H-bonds among single molecules.

Compound **4** crystallizes in the orthorhombic space group Pbc2 (1) with the density of 1.675 g·cm^−3^, which was consistent with the reported information^27^. The novel compound **1** and **2** can be crystallized from its oxidation reaction mother liquid respectively, both as colorless crystals.

The thermal stabilities of above-mentioned four compounds were determined by differential scanning calorimetry (DSC) and thermogravimetry (TG) measurements (with a heating rate of 10 °C·min^−1^). Crystal **4** exhibits a melting point of 143 °C and a decomposition peak temperature of 214 °C. Crystal **2** can be dehydrated after heating upto 90 °C and decomposes rapidly at 191.6 °C with explosion.

The rare gem-diol crystal **1** shows bad thermal stability. As shown in Fig. 6, the first endothermic process happened in the range of 90~107 °C in DSC curve, and with a mass loss of about 16.4% (cal:16.35%) in TG curve corresponding to elimination of two lattice water molecules, immediately followed by 1,2-elimination water reaction from gem-diol group in the 110.1~114.9 °C range (Fig. 6(b)). These two processes are happened almost at the same time based on the sustained mass loss shown on the TG curve.

Astonishingly, the crystal **1** dehydrated and underwent crystal-to-crystal solid transition once heating up to 130 °C as a stable new state. Then, perfect di(1H-tetrazol-5-yl)methanone (**3**) translucency crystal was formed. The crystal-to-crystal nature of this transition from **1** to **3** was confirmed firstly by X-ray single crystal diffraction.

Structurally, the existence of the strong electron-withdrawing tetrazole groups contributes a lot to the electrophilic activity of bridged methylene. Besides, we can observe from the DSC and TG curves that the gem-diol compound **1** becomes very unstable once losing crystalline water molecules. For a further understanding of crystal water’s influence on thermal stability of compound **1**, we have to study the nature of molecular packing structure (Fig. 5). The H-bonds between di(1H-tetrazol-5-yl)methanediol with crystal water molecules have very important contribution to the stability of crystal **1**.

Additionally, we can see a mushrooming number of hydrogen bonds throughout every unit which forms dense and infinite three-dimensional networks (Fig. 5b). Furthermore, each hydroxyl has a close relation with ubiquitous water molecules through H-bonds (Fig. 5c). The stability is further strengthened by multiple hydrogen bonds involving crystallization water molecules and by stacking interactions between adjacent ties (Figure S1). The results provide a better explanation for the phenomena that water molecules entail large barriers in dehydration decomposition of the gem-diol compound.

Herein we reported a facile green syntheses and characterizations of bis-tetrazole family including compounds 1, 2, 3, 4 as well as a mutual transformation between each other.

Compound 4 was completely converted to energetic salt compound 2 with excess potassium permanganate as oxidant, which represents a green and highly efficient synthetic route of methylene oxidation. Compound 1 and 3 were produced via the acidification of compound 2. The equilibrium distribution between 1 and 3 is connected with the pH of solution. The dehydration becomes barrierless in diluted hydrochloric acid. Noteworthily, compound 3 is a promising energetic compound as raw material of HEDMs. Besides, the stability of gem-diol compound 1 in atmosphere environment attributes to its dual role as either a donor or acceptor with crystal water resulting in the 3D hydrogen bonds nets.

An unprecedented crystal-to-crystal solid phase transition involving 1 and 3 was firstly confirmed. The mechanisms need to be further investigated. And the tetrazyl as electron withdrawing groups crystalline gem-diol (-C(OH)_2_-) compound, di(1H-tetrazol-5-yl)methanediol dihydrate (1, H_2_DTMdiol·2H_2_O), was obtained for the first time.

This research provides very significant experimental data for the methylene oxidation chemistry and alkanediol chemistry.

## Experimental

### Typical experiment procedures of the syntheses of compound 1, 2, 3 and 4

#### Di(1H-tetrazol-5-yl)methane (4, H_2_DTMane)

The synthesis of di(1H-tetrazol-5-yl) methane adapted from reference^28^ and obtained the crystal from isopropanol. IR (cm^−1^, KBr): 3008(s), 2863(s), 2736(s), 1747(m), 1560(s), 1375(s), 1324(s), 1197(m), 1072(s), 871(s). Elemental analysis (%) for C_3_H_4_N_8_ (152.12): C, 23.69; H, 2.65; N, 73.66; found: C, 23.57; H, 2.59; N, 73.58.

#### Potassium di(1H-tetrazol-5-yl)methanone dihydrate (2, [K(HDTMone)·2H_2_O]_n_)

A mixture of H_2_DTMane (50 mmol, 7.6 g) and potassium permanganate (110 mmol, 17.38 g) in water (500 mL) was stirred for 8 h at 80 °C. The mixture was cooled to the room temperature naturally, and colorless solution was collected by filtration. After concentrated the solution in vacuum condition, a water-soluble white powder ([K(HDTMone)·2H_2_O]_n_) was obtained, which could be recrystallized from water. IR (cm^−1^, KBr): 3636(s), 3493(s), 2393(s), 1955(s), 1676(s), 1491(s), 1388(s), 1344(s), 1164(m), 1115(s), 976(w), 923(s), 603(s), 494(w), 430(s). Elemental analysis (%) for C_3_H_5_N_8_O_3_K (240.25): C, 14.98; H, 2.08; N, 46.62; found: C, 15.13; H, 2.59; N, 46.99.

#### Di(1H-tetrazol-5-yl)methanone (3, H_2_DTMone)

Compound 2 (10 g) was dissolved in 100 mL of water. Under constant stirring, a solution of concentrated HCl in water was added dropwise. An insoluble white powder of which started instantly to precipitate, which was filtered and washed with ethanol to give compound 3. Lastly, the product was dried shortly under high vacuum as white power. (5.491 g, 79%). IR (cm^−1^, KBr): 3563(s), 3483(s), 2393(s), 1950(s), 1673(s), 1626(w), 1459(s), 1381(m), 1215(m), 1110(m), 1017(w), 907(s), 637(m),431(m). Elemental analysis (%) for C_3_H_2_N_8_O (166.13): C, 21.67; H, 1.20; N, 67.42; found: C, 21.73; H, 1.59; N, 67.69.

#### Di(1H-tetrazol-5-yl)methanediol dihydrate(1, H_2_DTMdiol·2H_2_O)

Compound 4 was stoichiometrically oxidized by potassium permanganate, and the filtrated reaction solution was acidified by HCl solution to pH = 2. After filtration, acidifying the solution, crystals of the product 1, used for X-ray structure analysis was obtained by culturing the filtrate for 10 days. IR (cm^−1^, KBr): 3408(s), 3123(s), 2949(s), 2858(s), 2696(s), 2590(s), 2443(s), 1797(m), 1678(m), 1557(s), 1476(s), 1438(m), 1357(s), 1241(s), 1140(s), 1094(s), 1034(m), 1011(m), 907(s), 819(s), 705(w), 564(m). Elemental analysis (%) for C_3_H_8_N_8_O_4_ (220.17): C, 16.35; H, 3.63; N, 50.87; found: C, 17.13; H, 3.59; N, 50.99.

## Electronic supplementary material

Supporting information

Dataset 2A

Dataset 2B

Dataset 3A

Dataset 3B

Dataset 1A

Dataset 1B
