# Supplementary material for: Gem-diol and Ketone Crystal-to-crystal Transition Phenomena
Source: Sci Rep. 2017 Oct 18;7:13426. doi: 10.1038/s41598-017-13596-6 (PMC5647396; doi:10.1038/s41598-017-13596-6)
Supplement: Supplementary file 1 — Supporting information [file 41598_2017_13596_MOESM1_ESM.doc]

**Supporting information to**

**Gem-diol and Ketone Crystal-to-crystal Transition Phenomena**

**Zhang Bo, Chen Sitong, Guo Weiming, Zhang Weijing, Wang Lin, Yang Li*, Zhang Jianguo**

(State Key Laboratory of Explosion Science and Technology, Beijing Institute of Technology, Beijing 100081, China)

^*^Correspondent author E-mail: yanglibit@bit.edu.cn, Tel & Fax +86-10-68911202

**Contents:**

**Table S1.** Crystal data and structure refinement details for the compound 1, 2, 3, 4

**Table S2~S10.** Selected bond lengths (Å) or bond angles (°) of compound 1, 2, 3, 4

**Table S11.** Selected hydrogen bonds of compound 4

**Table S12.** Detonation properties of compound 1, 3, 4

**Table S13.** Characteristic IR vibrations of the compound 1, 2, 3, 4

***Figure S1~S8.*** Crystal structures of compound 1, 2, 3, 4

***Figure S9.*** DSC curve of compound 2

***Figure S10.*** DSC and TG curves of compound 4

***Figure S11.***Powder X-ray diffraction (PXRD) curves of compound 1

***Figure S12.*** Powder X-ray diffraction (PXRD) curves of compound 3 (sample derived from compound 1)

**1. Materials and instruments**

**2. X-ray crystallography determinations**

**3. Detonation properties**

**4. Crystal structures**

**5. DSC and TG curves**

**6. Powder X-ray diffraction (PXRD)**

**7. FT-IR**

**8. References**

**1. Materials and instruments**

All the materials and chemical reagents with analytical grade were bought from the reagents company (Aladdin) and used without further purification. IR spectra of ligand were measured on a Bruker Equinox 55 infrared spectrometer by using KBr pellets from 400 to 4000 cm^-1^ with a resolution of 4 cm^-1^.

Elemental analyses (C, H and N) were performed on a Flash EA 1112 fully automatic trace element analyzer. The electron-microscope photos were taken on a Hitachi S4800 SEM.

The purities of the bulk samples were verified by powder X-ray diffraction (PXRD) measurements performed on a Bruker D8 advance diffractometer at 60 kV, 300 mA and Cu Kα radiation (λ=1.5406 Å), with a scan speed of 5°·min^-1^ and a step size of 0.02° in 2θ.

The single crystal X-ray diffraction data collections were carried out on a Rigaku AFC-10/Saturn 724+CCD diffractometer with graphite-monochromated Mo Kα radiation (λ=0.71073 Å) using the multi-scan technique. The structures were determined by direct methods using SHELXS-97 and refined by full-matrix least-squares procedures on F2 with SHELXL-97. All non-hydrogen atoms were obtained from the difference Fourier map and subjected to anisotropic refinement by full-matrix least squares on F2. Hydrogen atoms were obtained geometrically and treated as riding on the parent atoms or were constrained in the locations during refinements.

Thermal stability measurements were analyzed by using differential scanning calorimetry (DSC) on a CDR-4 of Shanghai Precision & Scientific Instrument Co., Ltd. at a heating rate of 10°C min^-1^from 50°C to 500°C. Thermogravimetric analyses (TG) were operated on Perkin–Elmer Pyris–1 thermogravimetric analyzer at a heating rate of 10°C·min^-1^ from 50°C to 500°C under dry oxygen-free nitrogen atmosphere with a flowing rate of 20 mL·min^-1^.

**2. X-ray crystallography determinations**

**Table S1.** Crystal data and structure refinement details for the compound **1, 2, 3, 4**

| Identification code | Compound **1** | Compound **2** | Compound **3** | Compound **4** |
| --- | --- | --- | --- | --- |
| CCDC | 1516736 | 1516733 | 1516734 | 1439053 |
| Empirical formula | C_3_H_8_N_8_O_4_ | C_3_H_5_N_8_O_3_K | C_3_H_2_N_8_O | C_3_H_4_N_8_ |
| Formula weight | 220.17 | 240.25 | 166.13 | 152.14 |
| Temperature/K | 153.15 | 153.15 | 153.15 | 153.15 |
| Crystal system | monoclinic | monoclinic | triclinic | orthorhombic |
| Space group | C2/c | P2_1_/m | P-1 | Pbc2(1) |
| a/Å | 11.752 | 6.8439(14) | 6.9602(14) | 6.870 |
| b/Å | 8.3925(17) | 18.244(4) | 9.6833(19) | 9.755(3) |
| c/Å | 9.0193(18) | 7.6545(15) | 9.754 | 18.008(6) |
| α/° | 90.00 | 90.00 | 104.42(3) | 90 |
| β/° | 92.41(3) | 111.11(3) | 90.03(3) | 90 |
| γ/° | 90.00 | 90.00 | 92.03(3) | 90 |
| Volume/Å^3^ | 888.8(3) | 891.6(3) | 636.2 | 1206.9(7) |
| Z | 4 | 4 | 4 | 8 |
| ρ_calc_/g.cm^-3^ | 1.645 | 1.790 | 1.734 | 1.675 |
| μ/mm^‑1^ | 0.147 | 0.602 | 0.141 | 0.128 |
| F(000) | 456.0 | 488.0 | 336.0 | 624 |
| Crystal size/mm^3^ | 0.26 × 0.18 × 0.11 | 0.26 × 0.24 × 0.12 | 0.12 × 0.1 × 0.04 | 0.63 x 0.11 x 0.10 |
| Radiation | Mo *K*α  (λ = 0.71073) | Mo *K*α  (λ = 0.71073) | Mo *K*α  (λ = 0.71073) | Mo *K*α  (λ = 0.71073) |
| 2Θfor data collection/° | 5.96 to 54.92 | 6.12 to 54.96 | 4.32 to 50 | 2.26 to 31.52 |
| Index ranges | -15 ≤ h ≤ 15  -10 ≤ k ≤ 10  -11 ≤ l ≤ 11 | -8 ≤ h ≤ 8  -23 ≤ k ≤ 23  -9 ≤ l ≤ 9 | -8 ≤ h ≤ 8  -11 ≤ k ≤ 11  -11 ≤ l ≤ 11 | -10≦h≦8,  -13≦k≦14,  -26≦l≦26 |
| Reflections collected | 6831 | 6379 | 8383 | 11275 |
| Independent reflections | 1011  [R_int_ = 0.0328,  R_sigma_ = 0.0161] | 2090  [R_int_ = 0.0264,  R_sigma_ = 0.0245] | 2237  [R_int_ = 0.1200,  R_sigma_ = 0.0900] | 2062  [R_int_ = 0.0399] |
| Data/restraints/parameters | 1011/0/69 | 2090/0/142 | 2237/0/218 | 2062 / 1 / 215 |
| Final R indexes  [I>=2σ (I)] | R_1_ = 0.0488,  wR_2_ = 0.1548 | R_1_ = 0.0348,  wR_2_ = 0.0797 | R_1_ = 0.1229,  wR_2_ = 0.3254 | R1 = 0.0457,  wR2 = 0.1284 |
| Final R indexes  [all data] | R_1_ = 0.0561,  wR_2_ = 0.1781 | R_1_ = 0.0367,  wR_2_ = 0.0845 | R_1_ = 0.1395,  wR_2_ = 0.3417 | R1 = 0.0507,  wR2 = 0.1408 |
| peak/hole / e Å^-3^ | 0.66/-0.64 | 0.38/-0.26 | 0.98/-0.70 | 0.293/-0.241 |

**Table S2.** Selected bond lengths (Å) of compound **1**

| Atom | Atom | Length/Å | Atom | Atom | Length/Å |
| --- | --- | --- | --- | --- | --- |
| O_1_ | C_2_ | 1.3917(19) | N_3_ | C_1_ | 1.330 |
| N_4_ | N_3_ | 1.346 | C_1_ | C_2_ | 1.519 |
| N_4_ | N_2_ | 1.300 | C_2_ | O_1_^1^ | 1.3917(19) |
| N_1_ | N_2_ | 1.358 | C_2_ | C_1_^1^ | 1.519 |
| N_1_ | C_1_ | 1.319 |  |  |  |

**Table S3.** Selected bond angles (°) of compound **1**

| Atom | Atom | Atom | Angle/˚ | Atom | Atom | Atom | Angle/˚ |
| --- | --- | --- | --- | --- | --- | --- | --- |
| N_2_ | N_4_ | N_3_ | 106.20(16) | O_1_ | C_2_ | O_1_^1^ | 114.2 |
| C_1_ | N_1_ | N_2_ | 105.65(15) | O_1_^1^ | C_2_ | C_1_ | 104.90(9) |
| C_1_ | N_3_ | N_4_ | 108.60(16) | O_1_^1^ | C_2_ | C_1_^1^ | 111.26(9) |
| N_4_ | N_2_ | N_1_ | 110.66(16) | O_1_ | C_2_ | C_1_^1^ | 104.90(9) |
| N_1_ | C_1_ | N_3_ | 108.89(17) | O_1_ | C_2_ | C_1_ | 111.26(9) |
| N_1_ | C_1_ | C_2_ | 125.44(15) | C_1_ | C_2_ | C_1_^1^ | 110.4 |
| N_3_ | C_1_ | C_2_ | 125.67(15) |  |  |  |  |

**Table S4.** Selected hydrogen bonds of compound **1**

| D | H | A | d(D-H)/Å | d(H-A)/Å | d(D-A)/Å | D-H-A/° |
| --- | --- | --- | --- | --- | --- | --- |
| O_2_ | H_2A_ | N_2_^1^ | 0.84 | 2.13 | 2.943 | 161.7 |
| O_2_ | H_2B_ | N_1_^2^ | 0.84 | 1.96 | 2.782 | 166.5 |
| O_1_ | H_1_ | O_2_^3^ | 0.84 | 1.87 | 2.694 | 168.2 |
| N_3_ | H_3_ | O_2_ | 0.89 | 1.88 | 2.759 | 168.5 |

**Table S5**. Selected bond lengths (Å) of compound **2**

| Atom | Atom | Length/Å | Atom | Atom | Length/Å |
| --- | --- | --- | --- | --- | --- |
| K_1_ | O_3_ | 2.7659(15) | K_2_ | N_7_^5^ | 3.0114(17) |
| K_1_ | O_3_^1^ | 2.7659(15) | K_2_ | N_7_^6^ | 3.0114(17) |
| K_1_ | O_4_ | 2.763 | O_1_ | K_1_^4^ | 3.0097(15) |
| K_1_ | K_2_ | 3.7614(14) | O_1_ | C_2_ | 1.222 |
| K_1_ | K_2_^2^ | 3.8275(12) | N_1_ | N_2_ | 1.333 |
| K_1_ | O_1_^3^ | 3.0097(15) | N_1_ | C_1_ | 1.342 |
| K_1_ | O_1_^2^ | 3.0097(15) | N_2_ | N_3_ | 1.325 |
| K_1_ | O_2_ | 2.861 | N_3_ | N_4_ | 1.341 |
| K_1_ | N_8_^3^ | 2.8460(18) | N_4_ | C_1_ | 1.332 |
| K_1_ | N_8_^2^ | 2.8460(18) | N_5_ | N_6_ | 1.320 |
| O_3_ | K_2_ | 2.8077(15) | N_5_ | C_3_ | 1.327 |
| O_4_ | K_2_^2^ | 2.850 | N_6_ | N_7_ | 1.327 |
| K_2_ | K_1_^4^ | 3.8275(12) | N_7_ | K_2_^7^ | 3.0114(17) |
| K_2_ | O_3_^1^ | 2.8077(15) | N_7_ | N_8_ | 1.320 |
| K_2_ | O_4_^4^ | 2.850 | N_8_ | K_1_^4^ | 2.8460(18) |
| K_2_ | O_1_^1^ | 2.9315(15) | N_8_ | C_3_ | 1.349 |
| K_2_ | O_1_ | 2.9315(14) | C_1_ | C_2_ | 1.482 |
| K_2_ | O_2_ | 2.727 | C_2_ | C_3_ | 1.492 |

**Table S6.**Selected bond angles (°) of compound **2**

| Atom | Atom | Atom | Angle/˚ | Atom | Atom | Atom | Angle/˚ |
| --- | --- | --- | --- | --- | --- | --- | --- |
| O_3_ | K_1_ | O_3_^1^ | 94.78(6) | O_3_^1^ | K_2_ | N_7_^5^ | 131.54(5) |
| O_3_^1^ | K_1_ | K_2_ | 48.03(3) | O_3_ | K_2_ | N_7_^5^ | 73.43(5) |
| O_3_ | K_1_ | K_2_ | 48.03(3) | O_3_^1^ | K_2_ | N_7_^6^ | 73.43(5) |
| O_3_^1^ | K_1_ | K_2_^2^ | 109.70(3) | O_3_ | K_2_ | N_7_^6^ | 131.54(5) |
| O_3_ | K_1_ | K_2_^2^ | 109.70(3) | O_4_^4^ | K_2_ | K_1_ | 174.86(5) |
| O_3_^1^ | K_1_ | O_1_^3^ | 145.65(4) | O_4_^4^ | K_2_ | K_1_^4^ | 46.06(4) |
| O_3_^1^ | K_1_ | O_1_^2^ | 73.90(4) | O_4_^4^ | K_2_ | O_1_ | 68.17(4) |
| O_3_ | K_1_ | O_1_^3^ | 73.90(4) | O_4_^4^ | K_2_ | O_1_^1^ | 68.17(4) |
| O_3_ | K_1_ | O_1_^2^ | 145.65(4) | O_4_^4^ | K_2_ | N_7_^6^ | 71.52(5) |
| O_3_ | K_1_ | O_2_ | 67.23(4) | O_4_^4^ | K_2_ | N_7_^5^ | 71.52(5) |
| O_3_^1^ | K_1_ | O_2_ | 67.23(4) | O_1_ | K_2_ | K_1_^4^ | 50.79(3) |
| O_3_ | K_1_ | N_8_^3^ | 89.43(5) | O_1_ | K_2_ | K_1_ | 108.90(3) |
| O_3_ | K_1_ | N_8_^2^ | 157.15(5) | O_1_^1^ | K_2_ | K_1_^4^ | 50.79(3) |
| O_3_^1^ | K_1_ | N_8_^3^ | 157.15(5) | O_1_^1^ | K_2_ | K_1_ | 108.90(3) |
| O_3_^1^ | K_1_ | N_8_^2^ | 89.43(5) | O_1_^1^ | K_2_ | O_1_ | 100.59(6) |
| O_4_ | K_1_ | O_3_ | 77.85(5) | O_1_ | K_2_ | N_7_^5^ | 137.91(5) |
| O_4_ | K_1_ | O_3_^1^ | 77.85(5) | O_1_^1^ | K_2_ | N_7_^5^ | 74.68(4) |
| O_4_ | K_1_ | K_2_ | 80.82(5) | O_1_ | K_2_ | N_7_^6^ | 74.68(4) |
| O_4_ | K_1_ | K_2_^2^ | 47.98(5) | O_1_^1^ | K_2_ | N_7_^6^ | 137.91(5) |
| O_4_ | K_1_ | O_1_^3^ | 68.15(4) | O_2_ | K_2_ | K_1_ | 49.22(5) |
| O_4_ | K_1_ | O_1_^2^ | 68.15(4) | O_2_ | K_2_ | K_1_^4^ | 79.57(5) |
| O_4_ | K_1_ | O_2_ | 127.02(7) | O_2_ | K_2_ | O_3_ | 68.51(5) |
| O_4_ | K_1_ | N_8_^2^ | 124.95(5) | O_2_ | K_2_ | O_3_^1^ | 68.51(5) |
| O_4_ | K_1_ | N_8_^3^ | 124.95(5) | O_2_ | K_2_ | O_4_^4^ | 125.64(7) |

**Table S7.** Selected bond lengths (Å) of compound **3**

| Atom | Atom | Length/Å | Atom | Atom | Length/Å |
| --- | --- | --- | --- | --- | --- |
| O1 | C2 | 1.217(6) | N9 | C4 | 1.353(7) |
| O2 | C5 | 1.210(6) | N10 | N11 | 1.287(7) |
| N1 | N2 | 1.301(6) | N11 | N12 | 1.372(6) |
| N1 | C1 | 1.374(7) | N12 | C4 | 1.312(7) |
| N2 | N3 | 1.346(6) | N13 | N14 | 1.312(6) |
| N3 | N4 | 1.314(6) | N13 | C6 | 1.310(7) |
| N4 | C1 | 1.305(7) | N14 | N15 | 1.331(7) |
| N5 | N6 | 1.366(6) | N15 | N16 | 1.326(6) |
| N5 | C3 | 1.316(7) | N16 | C6 | 1.349(7) |
| N6 | N7 | 1.288(7) | C1 | C2 | 1.454(7) |
| N7 | N8 | 1.329(6) | C2 | C3 | 1.489(7) |
| N8 | C3 | 1.334(7) | C4 | C5 | 1.470(7) |
| N9 | N10 | 1.330(7) | C5 | C6 | 1.481(7) |

**Table S8.** Selected bond angles (°) of compound **3**

| Atom | Atom | Atom | Angle/˚ | Atom | Atom | Atom | Angle/˚ |
| --- | --- | --- | --- | --- | --- | --- | --- |
| N_2_ | N_1_ | C_1_ | 105.0(5) | N_4_ | C_1_ | N_1_ | 112.5(5) |
| N_1_ | N_2_ | N_3_ | 107.0(4) | N_4_ | C_1_ | C_2_ | 126.5(5) |
| N_4_ | N_3_ | N_2_ | 112.4(5) | O_1_ | C_2_ | C_1_ | 121.5(5) |
| C_1_ | N_4_ | N_3_ | 103.1(4) | O_1_ | C_2_ | C_3_ | 119.3(5) |
| C_3_ | N_5_ | N_6_ | 106.6(5) | C_1_ | C_2_ | C_3_ | 119.2(5) |
| N_7_ | N_6_ | N_5_ | 109.5(5) | N_5_ | C_3_ | N_8_ | 107.4(5) |
| N_6_ | N_7_ | N_8_ | 107.1(5) | N_5_ | C_3_ | C_2_ | 130.8(5) |
| N_7_ | N_8_ | C_3_ | 109.3(5) | N_8_ | C_3_ | C_2_ | 121.8(5) |
| N_10_ | N_9_ | C_4_ | 108.8(5) | N_9_ | C_4_ | C_5_ | 120.5(5) |
| N_11_ | N_10_ | N_9_ | 107.0(5) | N_12_ | C_4_ | N_9_ | 107.9(5) |
| N_10_ | N_11_ | N_12_ | 110.4(5) | N_12_ | C_4_ | C_5_ | 131.6(5) |
| C_4_ | N_12_ | N_11_ | 105.9(5) | O_2_ | C_5_ | C_4_ | 119.6(5) |
| C_6_ | N_13_ | N_14_ | 102.7(4) | O_2_ | C_5_ | C_6_ | 121.9(5) |
| N_13_ | N_14_ | N_15_ | 114.0(5) | C_4_ | C_5_ | C_6_ | 118.5(5) |
| N_16_ | N_15_ | N_14_ | 104.8(5) | N_13_ | C_6_ | N_16_ | 112.1(5) |
| N_15_ | N_16_ | C_6_ | 106.4(5) | N_13_ | C_6_ | C_5_ | 126.5(5) |
| N_1_ | C_1_ | C_2_ | 121.0(5) | N_16_ | C_6_ | C_5_ | 121.4(5) |

**Table S9**. Selected bond lengths (Å) of compound **4**

| Bond | Lengths/ Å | Bond | Lengths/ Å |
| --- | --- | --- | --- |
| N(1)-C(1) | 1.337(4) | N(6)-N(7) | 1.296(3) |
| N(1)-N | 1.338(3) | N(7)-N(8) | 1.339(3) |
| N(1)-H(1) | 0.93(5) | N(8)-C(3) | 1.330(3) |
| N-N(3) | 1.296(3) | N(8)-H(8) | 0.87(4) |
| N(3)-N(4) | 1.353(4) | N(9)-C(4) | 1.342(3) |
| N(4)-C(1) | 1.329(3) | N(9)-N(10) | 1.344(3) |
| N(5)-C(3) | 1.317(3) | N(9)-H(9) | 0.99(5) |
| N(5)-N(6) | 1.356(4) | N(10)-N(11) | 1.306(3) |

**Table S10.** Selected bond angles (°) of compound **4**

| bond | angle／(°) | bond | angle／(°) |
| --- | --- | --- | --- |
| C(1)-N(1)-N | 108.9 | C(3)-N(5)-N(6) | 105.9 |
| C(1)-N(1)-H(1) | 126(3) | N(7)-N(6)-N(5) | 110.4 |
| N-N(1)-H(1) | 125(3) | N(6)-N(7)-N(8) | 106.4 |
| N(3)-N-N(1) | 106.1(3) | C(3)-N(8)-N(7) | 108.8 |
| N-N(3)-N(4) | 111.5 | C(3)-N(8)-H(8) | 124 |
| C(1)-N(4)-N(3) | 105.2 | N(7)-N(8)-H(8) | 127 |
| N(1)-C(1)-C | 125.1 | C(3)-C-H(2A) | 109.0 |
| H(2A)-C-H(2B) | 107.8 | N(8)-C(3)-C | 124.2 |
| N(4)-C(1)-N(1) | 108.4 | N(4)-C(1)-C | 126.5 |
| C(1)-C-C(3) | 112.8 | C(1)-C-H(2A) | 109.0 |
| C(1)-C-H(2B) | 109.0 | C(3)-C-H(2B) | 109.0 |
| N(5)-C(3)-N(8) | 108.5 | N(5)-C(3)-C | 127.4 |

**Table S11.** Selected hydrogen bonds of compound **4**

| D-H...A | d(D-H) | d(H...A) | d(D...A) | <(DHA) |
| --- | --- | --- | --- | --- |
| N(1)-H(1)...N(3)#1 | 0.89 | 1.95 | 2.813(3) | 164.2 |
| N(8)-H(8)...N(5)#2 | 0.89 | 2.04 | 2.890(3) | 160.0 |
| N(8)-H(8)...N(4)#2 | 0.89 | 2.61 | 3.171(3) | 122.0 |
| N(9)-H(9)...N(11)#2 | 0.89 | 1.95 | 2.808(3) | 162.7 |
| N(16)-H(16)...N(13)#1 | 0.89 | 2.06 | 2.899(3) | 157.8 |
| N(16)-H(16)...N(12)#1 | 0.89 | 2.59 | 3.106(3) | 117.9 |

**3. Detonation properties**

The heats of detonation (ΔH_det_) of compound 1, 3 and 4 were obtained on the basis of their enthalpy of formation, which were calculated by using the method density functional theory (DFT) code DMol_3_ b3lyp 6-311++g(d, p).Detonation properties including detonation velocity (*D*) and detonation pressure (*P*) of compound 1, 3 and 4 were obtained by using the empirical Kamlet-Jacobs formula (eq. 1-3)^[S1]^.

D=1.01Φ^1/2^(1+1 1.30ρ) (1)

P =1.558 Φρ^2^ (2)

Φ=31.68N(MQ)^1/2^ (3)

Where *D* is detonation velocity (km·s^-1^), *P* is detonation pressure (GPa), ρ is the density of explosive (g·cm^-3^), N is the moles of detonation gases per gram of explosive (mol·g^-1^), M is the average molecular weight of gaseous products (g·mol^-1^), Q is the heat of detonation (kcal·g^-1^). The detonation reactions are described as eq. 4~6. The calculated parameters are listed in Table S12.

C_3_H_8_N_8_O_4_(s) →3C(s) + 4H_2_O(l) + 4N_2_(g) (4)

C_3_H_2_N_8_O(s) →3C(s) + H_2_O(l) + 4N_2_(g) (5)

C_3_H_4_N_8_(s) →3C(s) + 4/3NH_3_(g) + 10/3N_2_(g) (6)

As in table S12, the detonation velocity (*D*) and detonation pressure (*P*) of compound 1, 3 and 4 were calculated to be 4< 1< 3< TNT< RDX, which may due to the ketone group benefits the increasing of crystal density and the gem-diol group along with water of crystallization make against the crystal density. The volume of gases after detonation (*V*) were 1< 3< 4, which indicates that the introduction of ketone group or gem-diol group is adverse to the enhancement of this parameter.

**Table S12.**Detonation properties of compound 1, 3, 4

| compound | ρ^a^ | Δ_f_*H*°_298_^b^ | *D*^c^ | *P*^d^ | *V*^e^ |
| --- | --- | --- | --- | --- | --- |
| 1 | 1.645 | -153.51 | 5802.40 | 14.13 | 407.2 |
| 3 | 1.734 | 150.21 | 6275.44 | 17.08 | 539.7 |
| 4 | 1.675 | 164.56 | 5335.93 | 12.08 | 687.5 |
| RDX | / | / | 8240 | 34.70 | / |
| TNT | / | / | 6950 | 22.05 | / |

a: lattice density, g·cm^-3^; b: enthalpy of formation, kJ·mol^-1^; c: detonation velocity, m·s^-1^; d: detonation pressure, GPa; e: volume of gases after detonation, L·kg^-1^.

**4. Crystal structures**


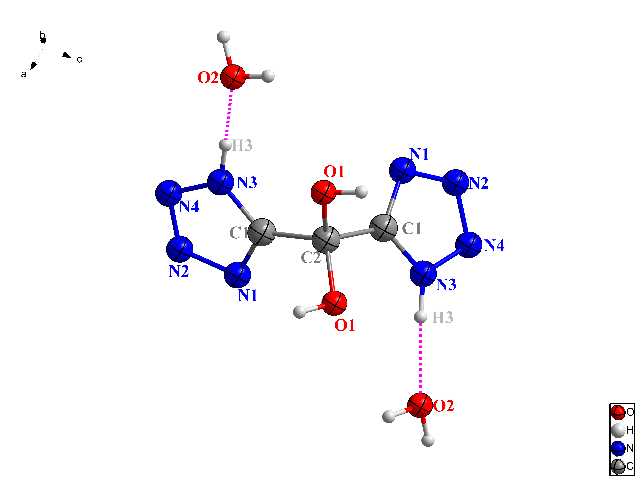


***Figure S1.*** Molecule structure of compound **1**


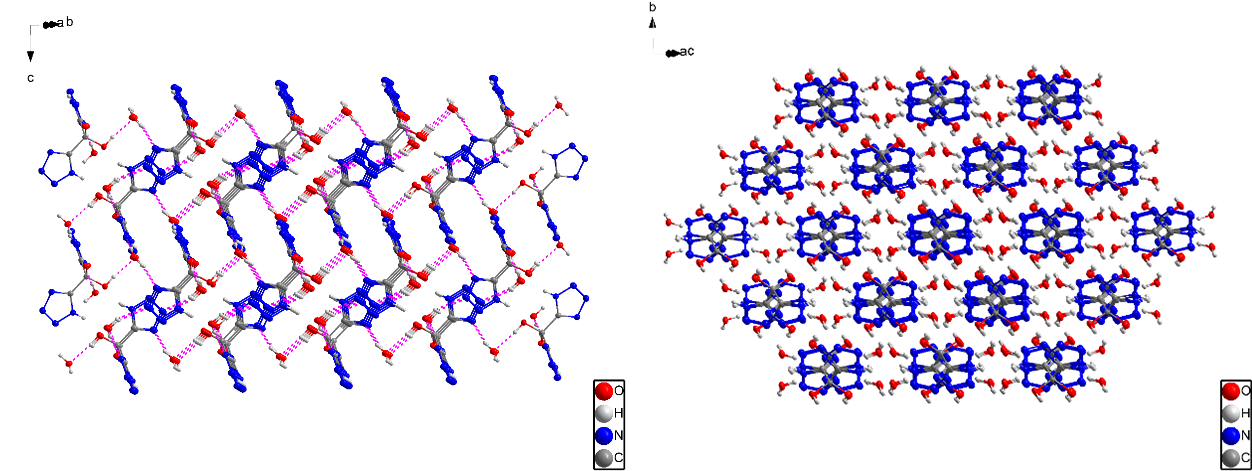


***Figure S2.*** Packing structure of **1** along different viewing directions (1 column picture)


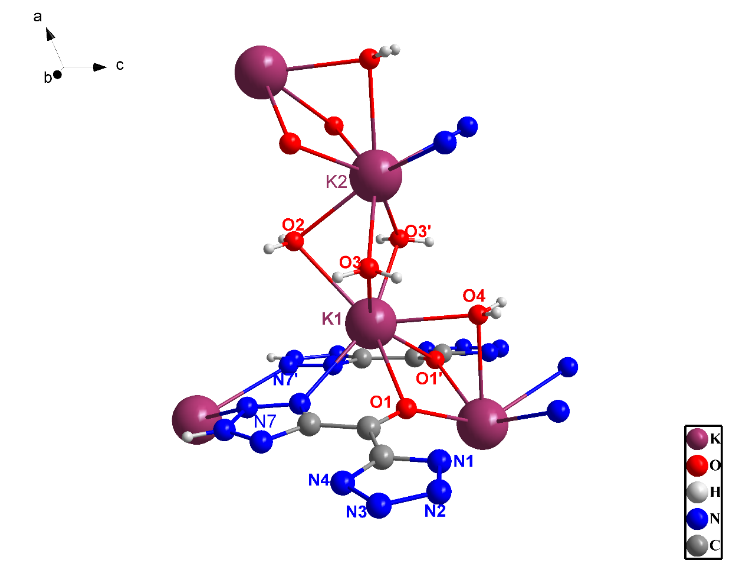


***Figure S3.*** Molecule structure of compound **2**


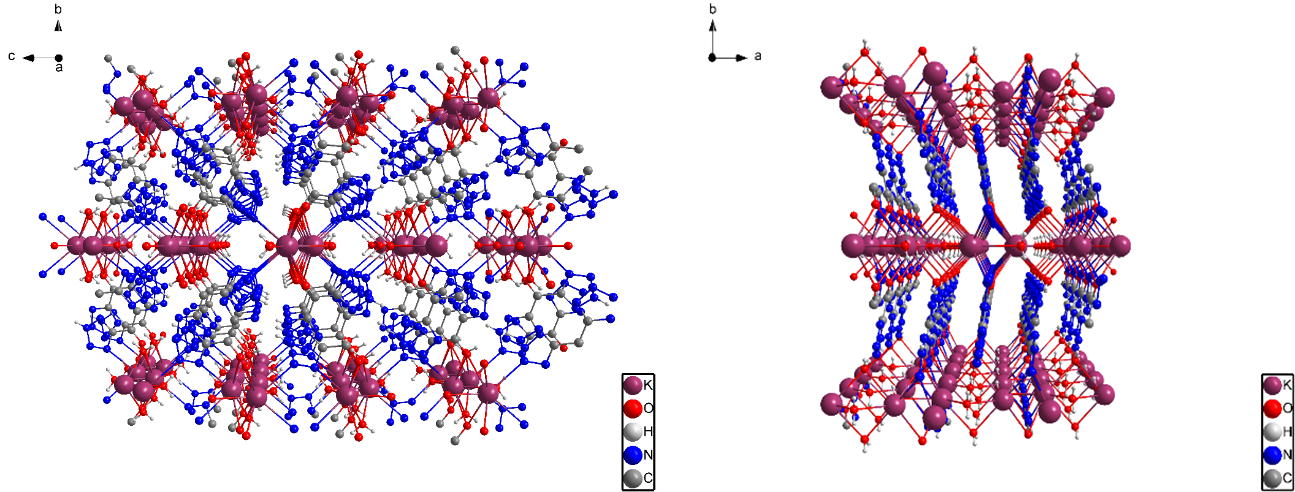


***Figure S4.*** Packing structure of **2** along different viewing directions (1 column picture)


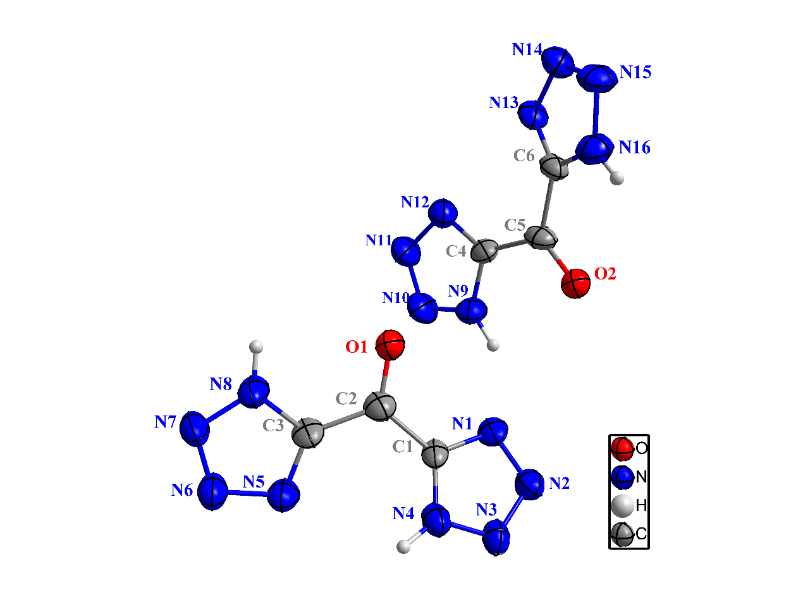


***Figure S5.*** Molecule structure of compound **3**


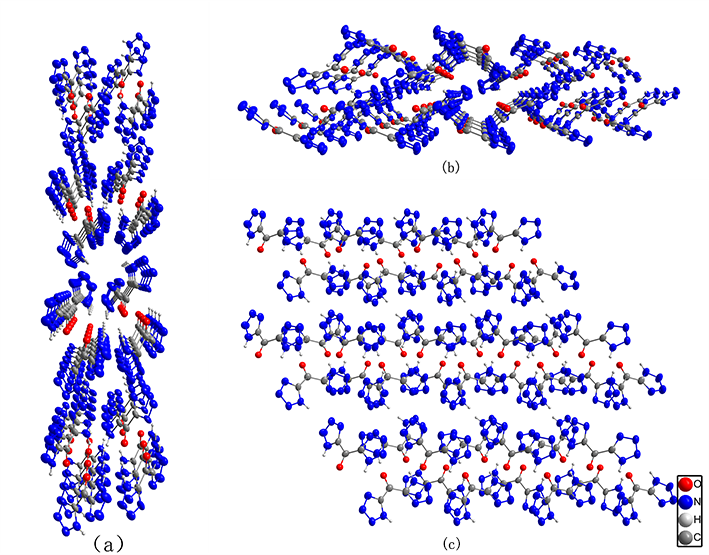


***Figure S6.*** Packing structure of **3** along different viewing directions (1 column picture)


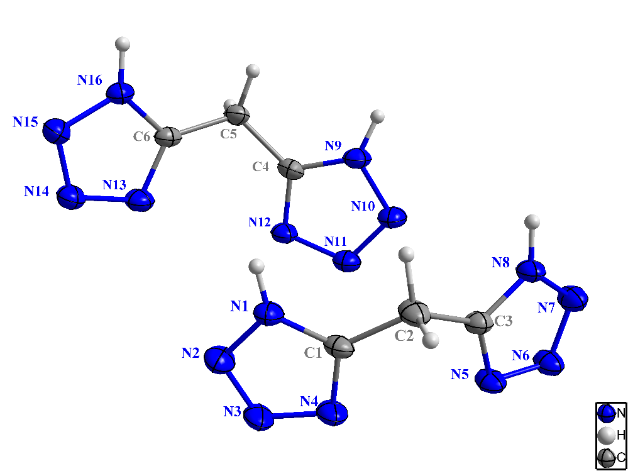


***Figure S7.*** Molecule structure of compound **4**


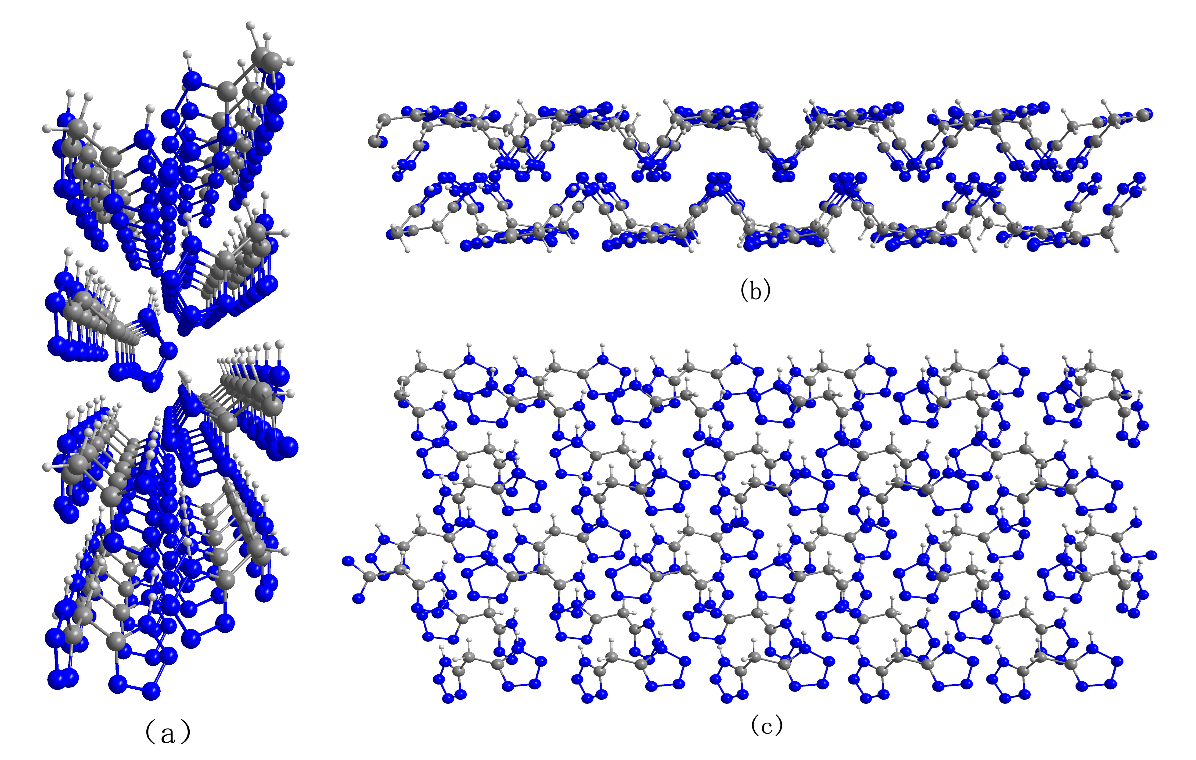


***Figure S8.*** Packing structure of **4** along different viewing directions (1 column picture)

**5. DSC and TG curves**

***Figure S9.*** DSC curve of compound **2**

***Figure S10 .***DSC and TG curves of compound **4**

**6. Powder X-ray diffraction (PXRD)**

**
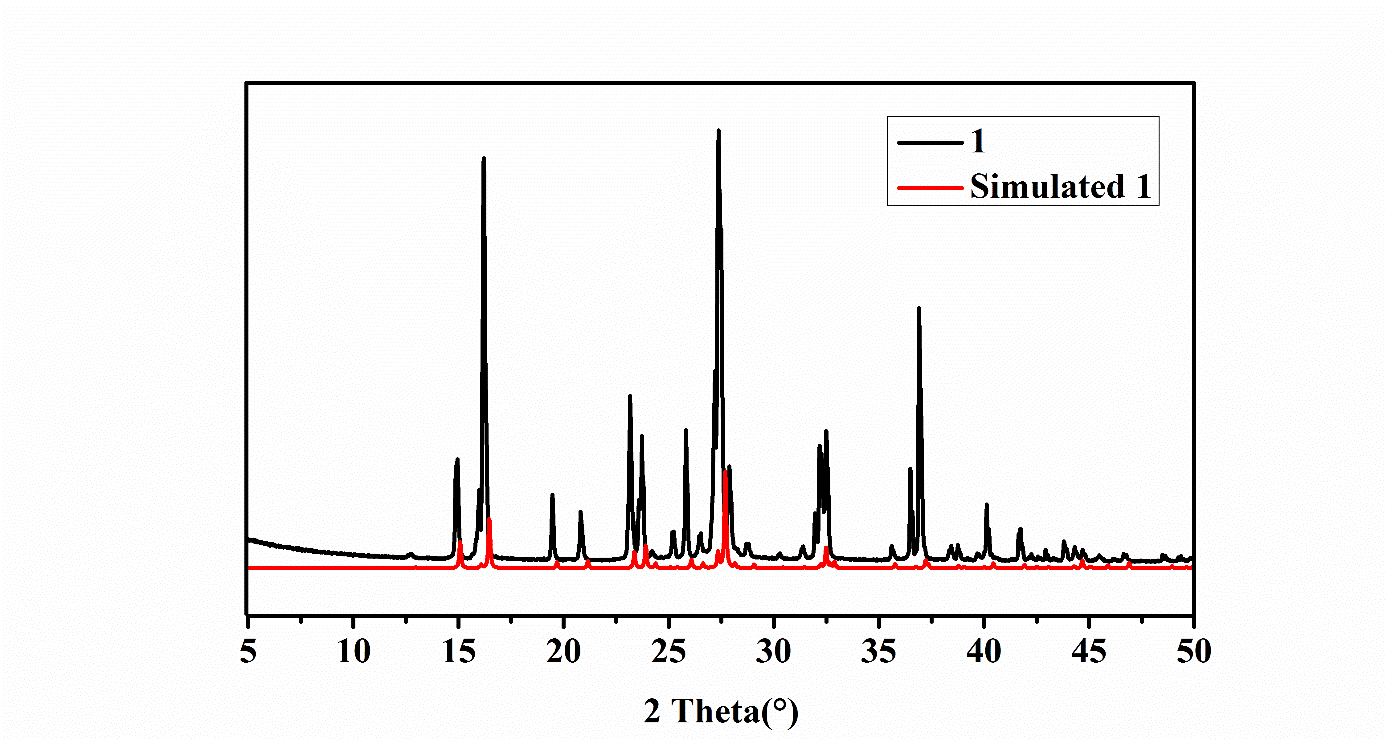
**

***Figure S11 .***Powder X-ray diffraction (PXRD) curves of compound 1

**
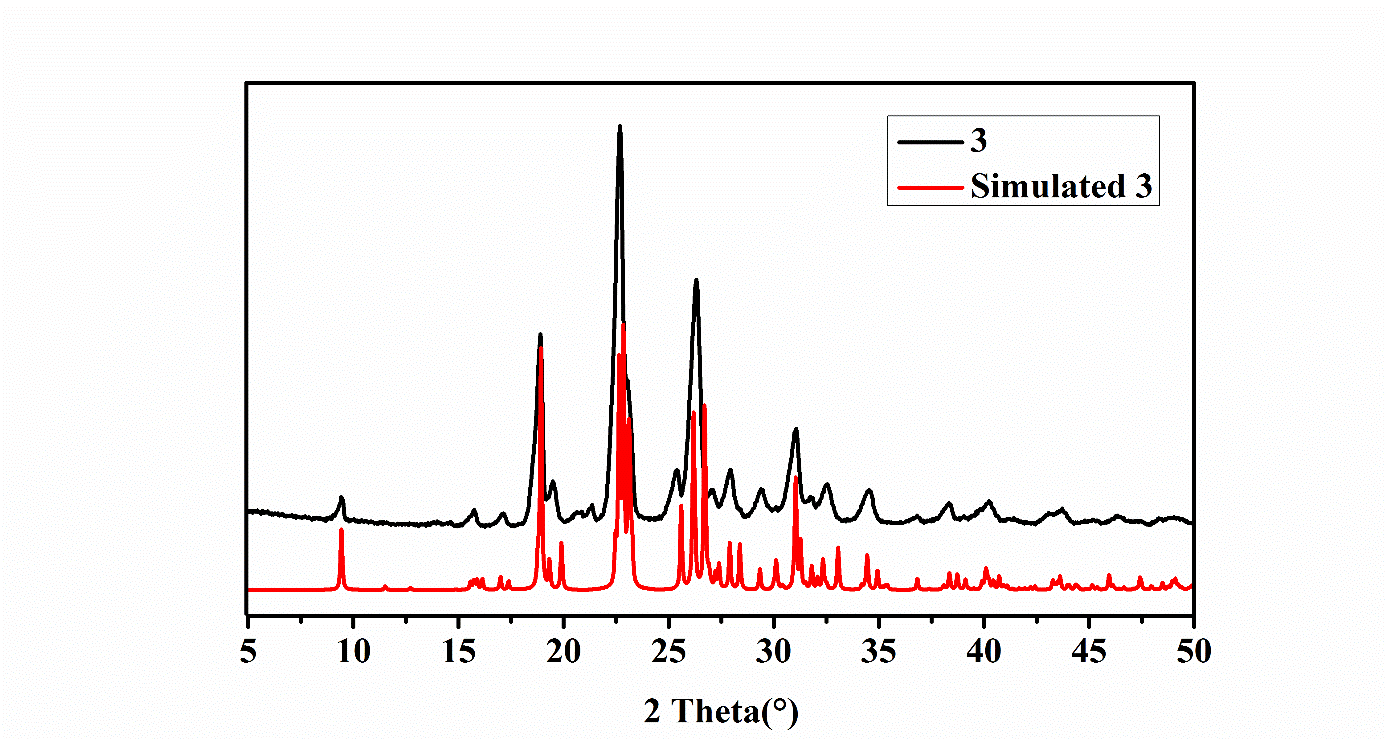
**

***Figure S12.***Powder X-ray diffraction (PXRD) curves of compound 3 (sample derived from compound 1)

**7. FT-IR**

The characteristic IR vibrations of the compound 1, 2, 3, 4 are listed in table S13, which shows C-H vibrations at 2863(s) and 1375(s) of compound 4, C-O vibrations at 1140(s) of compound 1 and C=O vibrations at of 1676(s) and 1673(s) of compound 2 and 3 respectively. As for vibrations in C-C bonds, the peaks of compound 1 distinctly increase by at least two contrast to compound 2, 3 or 4, which may due to the influence of the gem-diol group. And the peak around 2400, one of the vibrations in tetrazoles, of compound 4 is too weak to be recognized.

**Table S13.** Characteristic IR vibrations of the compound 1, 2, 3, 4 (cm^-1^)

| Compound 1 | Compound 2 | Compound 3 | Compound 4 |  |
| --- | --- | --- | --- | --- |
| **/** | **/** | **/** | 2863(s), 1375(s) | C-H |
| 3408(s), 1357(s) | 3493(s) | **/** | **/** | O-H |
| 1140(s) | **/** | **/** | **/** | C-O |
| **/** | 1676(s) | 1673(s) | **/** | C=O |
| 1241(s), 1094(s), 1034(m), 1011(m) | 1164(m), 1115(s) | 1215(m), 1110(m) | 1197(m) | C-C |
| 2443(s), 1797(m), | 2393(s), 1955(s) | 2393(s), 1950(s), | 1747(m), | tetrazole |

**8. References**

[S1] Kamlet, M. J., Jacobs, S., Chemistry of Detonations. I. A Simple Method for Calculating Detonation

Properties of C–H–N–O Explosives. *J. Chem. Phys.* **48**, 23-35 (1968).
